# Prognostic value of c-MET in oesophageal squamous cell carcinoma: a study based on the mRNA expression in TCGA database and a meta-analysis

**DOI:** 10.3389/fmed.2025.1548160

**Published:** 2025-02-26

**Authors:** Qiqi Zhang, Xiujuan Li, Jian Li, Zhiqiang Zhang

**Affiliations:** ^1^Department of Gastroenterology II, The First Affiliated Hospital of Xinjiang Medical University, State Key Laboratory of Pathogenesis, Prevention and Treatment of High Incidence Diseases in Central Asia, Urumqi, China; ^2^Department of Pathophysiology, School of Basic Medical Sciences, Xinjiang Medical University, Urumqi, China

**Keywords:** proto-oncogene proteins mesenchymal-epithelial transition factor, oesophageal squamous cell carcinoma, prognosis, meta-analysis, biomarkers

## Abstract

**Objective:**

This study aims to assess the mesenchymal-epithelial transition factor’s (c-MET) prognostic value in oesophageal carcinoma (ESCA) through a meta-analysis and bioinformatics.

**Methods:**

We analysed c-MET expression in ESCA tissues using data from The Cancer Genome Atlas (TCGA) and conducted a meta-analysis to evaluate its association with clinicopathological factors and survival outcomes. The meta-analysis included studies reporting hazard ratios (HRs) and odds ratios (ORs) for survival and metastatic outcomes.

**Results:**

The Cancer Genome Atlas analysis revealed elevated c-MET expression in ESCA, which was significantly correlated with lymph node metastasis, tumour grade and stage, though not with overall survival (OS). In the meta-analysis, 278 publications were identified, and 89 duplicates were removed. After screening, 176 articles were excluded, leaving 13 for full-text review. Of these, 5 studies lacked sufficient survival data, resulting in 8 eligible studies with a total of 1,488 patients. Meta-analysis findings indicated that high c-MET expression was associated with worse OS (HR = 1.54, 95% confidence interval [CI]: 1.17–2.01; *p* = 0.002), distant metastasis (OR = 1.97, 95% CI: 1.14–3.40; *p* = 0.02) and advanced stage (OR = 2.23, 95% CI: 1.41–3.53; *p* = 0.0006).

**Conclusion:**

High c-MET expression is associated with poor prognosis and advanced disease in ESCA, highlighting its potential as a biomarker for risk stratification. Further studies are needed to confirm its prognostic value and explore therapeutic implications.

## 1 Introduction

Oesophageal carcinoma (ESCA) is one of the deadliest malignancies globally, with a significant increase in incidence. According to the IARC, 604,100 new ESCA cases and 544,076 deaths were reported in 2020 (1). Oesophageal carcinoma ranks seventh in incidence and sixth in mortality worldwide. Oesophageal squamous cell carcinoma (ESCC), the most prevalent histological type, accounts for approximately 90% of ESCA cases (2). Treatment options including surgery, radiotherapy, chemotherapy, endoscopy and traditional Chinese medicine have been implemented widely; however, the 5-year survival rate for patients with ESCA remains poor (3). The increasing incidence and low survival rate underscore the urgent need for improved diagnostic and predictive biomarkers.

The mesenchymal-epithelial transition factor (c-MET), a receptor tyrosine kinase found on the surface of various epithelial cells, typically plays a crucial role in wound recovery and tissue remodelling in humans. However, abnormal activation of the c-MET signaling pathway often occurs during cancer development, promoting the growth, invasion and metastasis of tumour cells (4). Numerous studies have confirmed that c-MET is overexpressed or mutated in various solid tumours, including lung, gastric, liver, breast, skin and colorectal cancers, with significant effects on tumour formation and progression (5, 6). The activation of c-MET is frequently associated with high-grade and advanced-stage tumours. The overexpression of c-MET has been shown to correlate with pathological stage, tumour grade, muscle invasion and lymph node involvement in bladder cancer (7). High c-MET expression is linked to an increased risk of lymph node metastasis in various tumours (8–10). Activation of c-MET has also been associated with tumour angiogenesis. By activating downstream signaling pathways, c-MET promotes the proliferation and migration of vascular endothelial cells, thereby supporting tumour growth and metastasis (11, 12). Mesenchymal-epithelial transition with different mutations can have varying effects; METex14 tumours exhibit differences in immuno-oncology biomarkers and the somatic landscape compared with non-METex14 NSCLC tumours, with variations in immune profiles potentially influencing immunotherapy selection in MET-altered NSCLC (13). Germline or somatic mutations, chromosomal rearrangements, gene amplification, transcriptional upregulation in MET or alterations in autocrine or paracrine c-MET signalling have been associated with cancer cell proliferation and survival, including in renal cell carcinoma, and linked to disease progression (14). Recently, small molecule inhibitors targeting c-MET, such as capmatinib and tepotinib, have entered clinical trials and shown positive results in patients with non-small cell lung cancer (15). Nonetheless, the specific role of c-MET in ESCC has not been well documented. Although earlier studies have identified c-MET expression in ESCC, its prognostic value and therapeutic potential as a therapeutic target remain unclear, mainly due to differences in methodology and research priorities across studies, which have led to divergent understandings of c-MET’s action.

Addressing the unmet need in ESCC management, it is crucial to explore the clinical significance of c-MET and its association with patient prognosis. Further investigation into c-MET expression in ESCC and its correlation with clinical outcomes could provide valuable insights for future clinical applications. We aimed to assess the prognostic impact of c-MET expression in ESCC, contributing to the foundation of potential therapeutic strategies and advancing clinical practise in this challenging area.

## 2 Materials and methods

### 2.1 UALCAN and GEPIA online cancer data analysis

The expression level of c-MET mRNA and its prognostic value in ESCA can be assessed by analysing data from The Cancer Genome Atlas (TCGA) database. This analysis can be performed using two online tools: UALCAN^1^ and GEPIA.^2^ The comprehensive cancer data research platform UALCAN offers convenient access to multiple public cancer omics datasets, including TCGA, MET500, CPTAC and CBTTC (16). In contrast, the GEPIA database integrates research data from TCGA and GTEx projects, covering RNA sequencing information from more than 9,736 tumour samples and 857 normal samples. Using these tools, researchers have been able to investigate the expression pattern of c-MET in oesophageal cancer and its impact on prognosis (17).

### 2.2 Literature searching

A comprehensive literature search was conducted according to the PRISMA guidelines (18) to identify studies published up to September 2022. The following databases were searched: PubMed, Web of Science, Cochrane Library, Wanfang and CNKI. The search strategy included the following key phrases: ‘esophageal squamous cell carcinoma’ OR ‘oesophageal squamous cell carcinoma’ OR ‘esophageal carcinoma’ OR ‘ESCC’ OR ‘ESCA’ AND ‘c-MET’ OR ‘MET’ OR ‘hepatocyte growth factor receptor’ OR ‘HGFR’.

For each database, the search strategy was tailored to fit the specific syntax and indexing terms used by the database. For example, in PubMed, the search query was constructed as follows: (‘esophageal squamous cell carcinoma’ [MeSH Terms] OR ‘oesophageal squamous cell carcinoma’ [MeSH Terms] OR ‘esophageal carcinoma’ [MeSH Terms] OR ‘ESCC’ [All Fields] OR ‘ESCA’ [All Fields]) AND (‘c-MET’ [MeSH Terms] OR ‘MET’ [MeSH Terms] OR ‘hepatocyte growth factor receptor’ [MeSH Terms] OR ‘HGFR’ [All Fields]). In Web of Science and Scopus, the search was conducted using the ‘Topic’ or ‘Title/Abstract/Keywords’ fields to ensure broad coverage. The search terms were combined using Boolean operators (AND/OR) to refine the search results. The search results were then exported to reference management software (e.g., EndNote) for deduplication and initial screening.

To enhance the reproducibility of this study, the search was restricted to titles and abstracts and supplemented by manual searches of reference lists from relevant articles. Searches were limited to studies in English and Chinese, and all identified articles were evaluated for eligibility through a multi-step screening process. The search timeframe extended up to September 2022, including studies from all years prior, to ensure comprehensive coverage of existing literature.

### 2.3 Selection criteria

The inclusion and exclusion process was conducted independently by two reviewers to ensure accuracy and minimise bias. Studies included in the meta-analysis had to meet the following conditions: (1) histopathological confirmation of ESCC was required for the study participants; (2) immunohistochemistry or fluorescence *in situ* hybridisation (FISH) techniques were used to assess c-MET protein expression in the study; (3) the relationship between c-MET expression and DSS, DFS, PFS or overall survival (OS) was investigated; and (4) the full text of the study had to be available for review. If the study did not utilise Kaplan–Meier survival curves for analysis or if data were incomplete and hazard ratios (HRs) could not be calculated, the study was excluded. Two reviewers independently screened articles based on titles and abstracts and then reviewed the full text to determine if inclusion criteria were met. During the review, disagreements between reviewers were resolved by discussion; if no agreement could be reached, a third reviewer was invited to participate in the discussion to ensure rigour and transparency throughout the selection process.

### 2.4 Data extraction and quality assessment

Literature was initially screened by reading the title and abstract, and the uncertain literature was determined by further reading the full text. Literature selection was completed by two investigators independently, and when opinions were inconsistent, they were resolved through discussion or consultation with the third investigator. To assess the scientific quality of the included studies, we used the Newcastle-Ottawa Scale (NOS). This scale is designed to evaluate the quality of randomised, case–control and cohort studies and scored by examining the selection, comparability and assessment of exposure or outcomes of the study participants. The NOS uses a semiquantitative scoring system with a maximum total score of nine, of which six and above are considered high-quality studies. For each article, the following details were extracted: first author’s name, publication year, article title, sample size, study site, tumour type, clinicopathological characteristics (including number of patients by sex, median or mean age, TNM stage, distant metastasis, tumour differentiation and clinical stage), c-MET protein expression detection technique, the criteria for high expression determination and survival data (including HRs for OS and its 95% confidence interval [CI]).

### 2.5 Statistical analysis

Review Manager 5.3 software (RevMan, Cochrane Collaborative, Oxford, UK) was used, which graphically presented results and facilitated meta-analysis. To assess heterogeneity across studies, the Q test and I^2^ statistic were used based on chi-squared (χ^2^) statistics. Significant heterogeneity amongst studies was considered if *p* < 0.05 in the Q test and *I*^2^ > 50%. If homogeneity across studies was confirmed, a fixed-effects model was used to calculate the combined treatment effect and HR with its 95% CI. If heterogeneity amongst studies was significant, a random-effects model was used instead. Additionally, funnel plots and Egger’s test were used to assess publication bias, with *p* < 0.1 considered statistically significant, indicating publication bias.

## 3 Results

### 3.1 C-MET mRNA expression was up-regulated in ESCA

**Figure 1:**
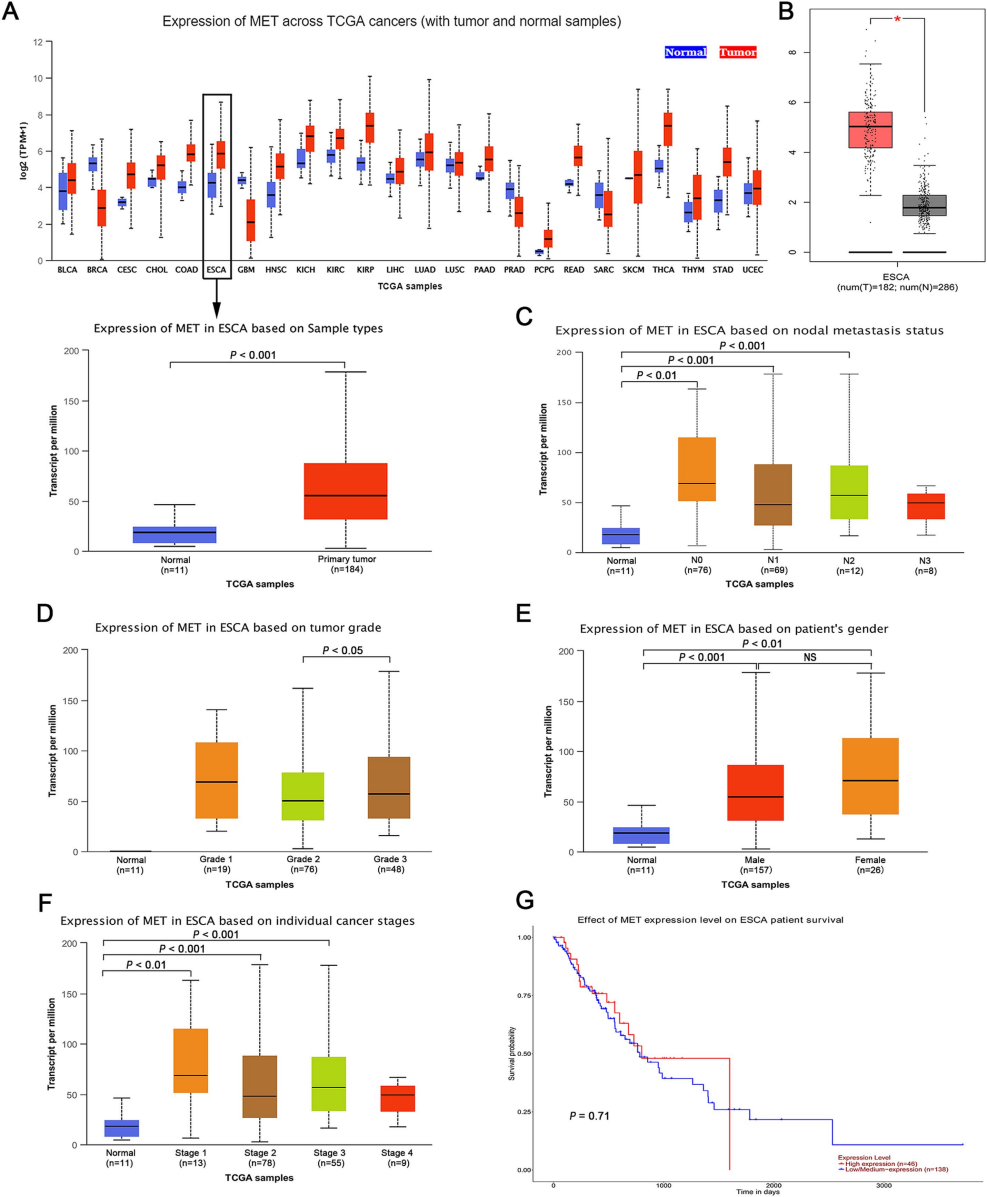
Expression level of c-MET mRNA in ESCA. **(A)** c-MET mRNA expression was remarkably overexpressed in ESCA tissues compared with normal peritumoral tissues in TCGA database. **(B)** The expression of c-MET mRNA in ESCA tissues was significantly overexpressed compared with that in normal peritumoral tissues by GEPIA (*p < 0.05). **(C)** The mRNA expression level of c-MET in different lymph node metastasis status based on TCGA database. **(D)** The mRNA expression level of c-MET in different tumor grades based on TCGA database. **(E)** The mRNA expression level of c-MET in different genders based on TCGA database. **(F)** The mRNA expression level of c-MET in different cancer stages based on TCGA database. **(G)** OS of ESCA patients in c-MET-low/medium expression group and c-MET-high expression group based on TCGA database. c-MET, cellular-mesenchymal epithelial transition factor; ESCA, esophageal carcinoma; TCGA, the cancer genome atlas; GEPIA, gene expression profiling interactive analysis; OS, overall survival.

By analysing the public cancer database TCGA, we investigated c-MET mRNA expression levels in adjacent and tumour tissues from patients with ESCA. The results of the analysis showed that c-MET mRNA expression was higher in tumour tissues than in adjacent non-cancerous tissues in most cases, particularly in oesophageal cancer, and this difference was statistically significant (*p* < 0.001; Figure 1A). In addition, this finding was validated by the GEPIA database, which integrated datasets from TCGA and GTEx and included more oesophageal cancer samples, further confirming differences in c-MET mRNA expression between tumours and adjacent non-cancerous tissues (*p* < 0.05; Figure 1B). Furthermore, we investigated the association of c-MET mRNA expression with patient characteristics in ESCA samples using data from TCGA database. As shown in Figures 1C–F, the expression level of c-MET mRNA was associated with lymph node metastasis, tumour grade and stage but not significantly with the gender of the patients (Figures 1E, F). Finally, we analysed the relationship between c-MET mRNA expression in TCGA database and the prognosis of patients with ESCA and found no significant difference in survival time between patients with high c-MET expression and those with low or moderate c-MET expression (*p* = 0.71; Figure 1G).

### 3.2 Identification and characterisation of relevant studies

**Figure 2:**
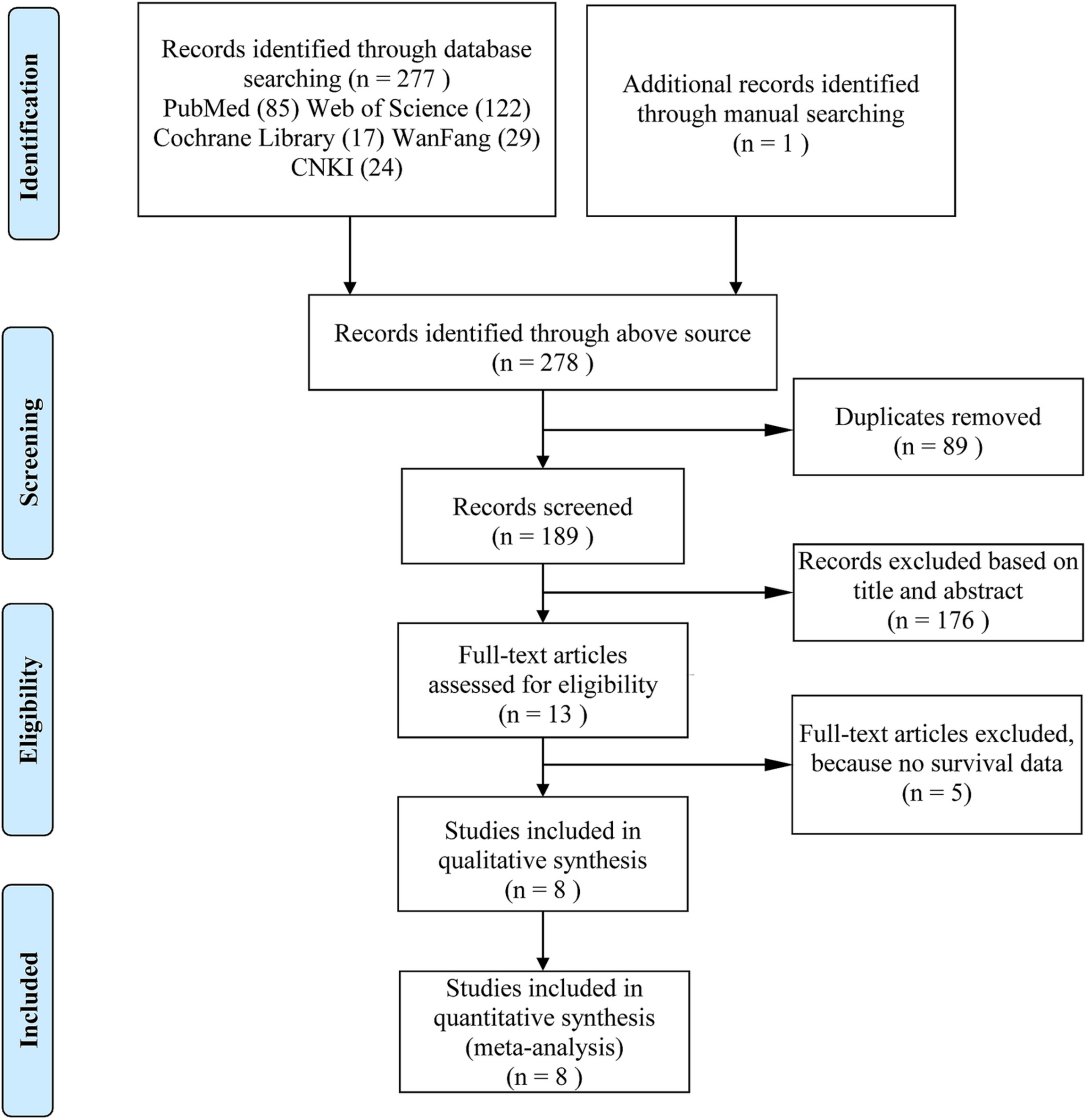
Flowchart of the literature searching in this meta-analysis.

To further investigate the association between c-MET expression and ESCA prognosis, we performed a meta-analysis. A total of 278 relevant articles were initially identified through databases and hand searches. After removing 89 duplicate publications, we excluded 176 articles by reviewing the titles and abstracts. Subsequently, we read and assessed the full text of the remaining 13 studies. Unfortunately, five studies (19–23) were excluded because they lacked the necessary survival data for the analysis, and a total of eight publications (24–31) eventually met the inclusion criteria. Figure 2 shows the literature screening process in detail. The total number of patients included in the meta-analysis was 1,488, with a mean sample size of 186, ranging from 90 to 495. Literature quality was assessed using the NOS, and the mean score of the included studies was 7.25 points (score range: 6–8 points), indicating high study quality. We extracted HRs and 95% CIs from the 8 articles that met the criteria. Table 1 presents the main characteristics of these studies.

**Table 1:** Main characteristics of the 8 included studies in the meta-analysis.

Study year	Country	Cancer type	Technology	Sample size	Median or mean age (y)	Gender (F/M)	c-MET (H/L)	Median or mean follow-up time (months)	Outcome	HR (95%CI)	*p* value	Cut-off value	NOS score
Hara 2019	Japan	ESCC	IHC	147	66	25/122	73/74	NA	OS	1.79 (1.03–3.04)	*p* = 0.038	H-score ≥ 90	7
Kim 2016	South Korea	ESCC	IHC	200	65 (41–83)	12/188	42/158	33.2 (0.6–178.7)	OS	1.12 (0.73–1.72)	*p* = 0.601	H-score ≥ 50	7
Ozawa 2015	Japan	ESCC	IHC	104	64	18/86	72/32	NA	OS	2.237 (1.066–5.190)	*p* = 0.017	H-score ≥ 40	7
Shi 2022	China	ESCC	IHC	172	61.8 (32–83)	54/118	98/74	34 (2–108)	OS	0.371(0.237–0.582)	*P* < 0.001	IHC Score > 4	8
Wang 2019	China	ESCC	FISH	495	61 (34–83)	87/408	28/467	35 (3–102)	OS	1.926 (1.243–2.983)	*p* = 0.003	Copy number ≥ 5 and a MET/CEP7 ratio ≥ 2	8
Xu 2015	China	ESCC	IHC	90	59	17/73	39/51	26.3	OS	1.805(1.045–3.117)	*p* = 0.034	H-score ≥ 20	8
Xu 2016	China	ESCC	IHC	180	59 (37–80)	16/164	84/96	46.4	OS	0.459(0.287–0.733)	*p* = 0.001	H-score ≥ 160	7
Zhou 2016	China	ESCC	IHC	100	59	33/67	49/51	NA	OS	2.34 (1.63–4.45)	*p* < 0.05	NA	6

F, female; M, male; H, high; L, low; HR, hazard ratio; CI, confidence interval; NOS, Newcastle-Ottawa Scale; ESCC, esophageal squamous cell carcinoma; IHC, immunohistochemistry; NA, not availiable; OS, overall survival; FISH, Fluorescence in situ hybridization.

### 3.3 Relationship between c-MET expression and ESCC survival

**Figure 3:**
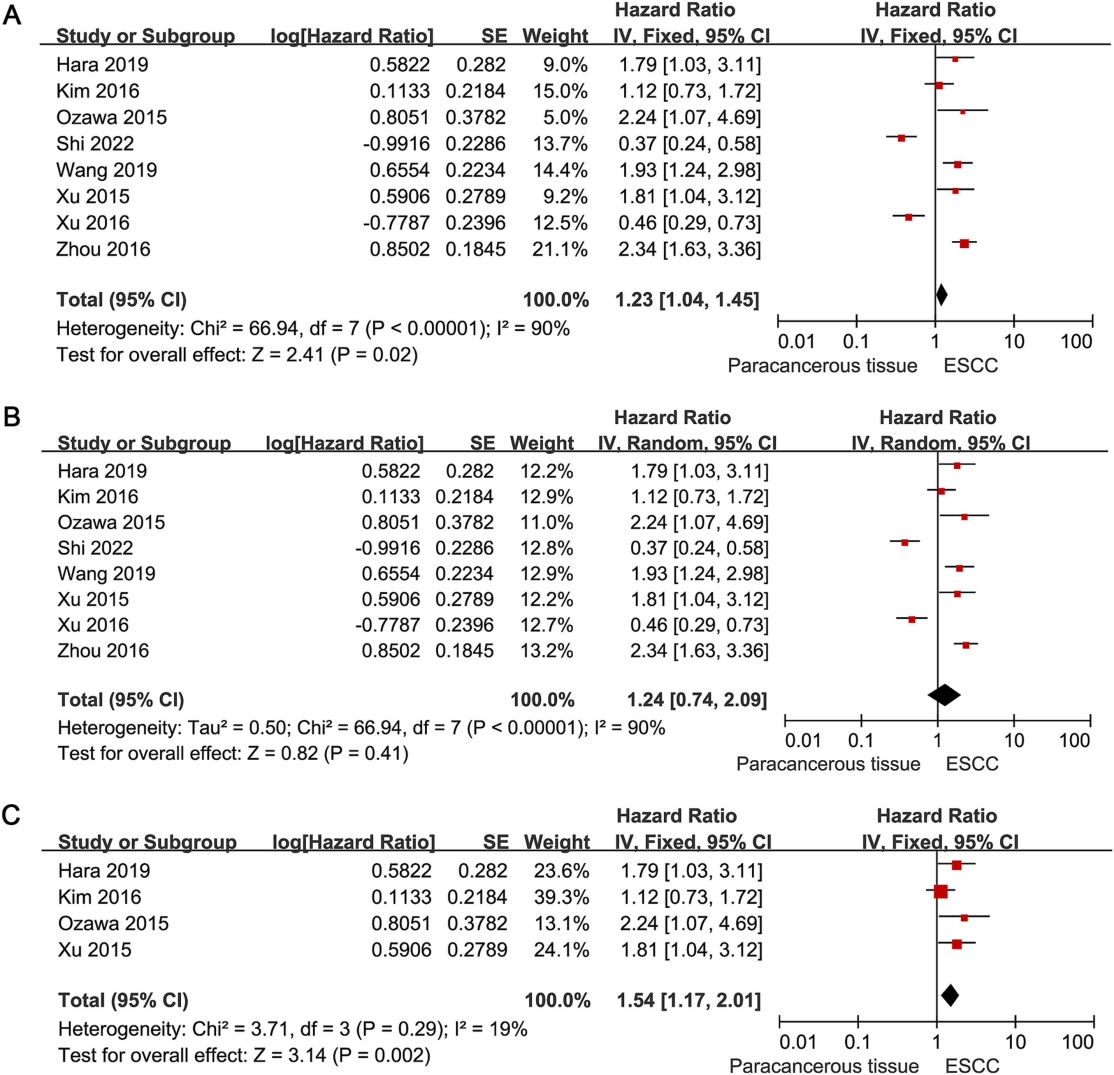
Forest plots of HRs for the association between c-MET expression and OS in patients with ESCC. Forest plots of the overall association between c-MET expression and OS in ESCC were performed using **(A)** fixed effect model and **(B)** random effect model. **(C)** After excluding some of the included literatures, forest plot of the overall association between c-MET expression and OS in ESCC was performed using fixed effect model. HRs, hazard ratios; c-MET, cellular-mesenchymal epithelial transition factor; OS, overall survival; ESCC, esophageal squamous cell carcinoma; CI, confidence interval.

In the collated dataset, a total of six studies provided data on OS, whereas two studies (27, 28) reported both OS and DFS. Therefore, we used OS as the primary outcome to evaluate the effect of c-MET expression levels on survival in patients with ESCC. We first analysed the relationship between c-MET expression and OS in eight studies. Preliminary analysis showed that patients with high c-MET expression had a worse prognosis than those with low c-MET expression (HR = 1.23, 95% CI: 1.04–1.45; *p* = 0.02; Figure 3A). However, because the I^2^ value exceeded 50% in the heterogeneity test, we reperformed the analysis using a random-effects model. The results of this analysis showed no significant correlation between c-MET expression levels and OS in patients ESCC (HR = 1.24, 95% CI: 0.74–2.09; *p* = 0.41; Figure 3B).

Given that the cut-off value, antibody and technical method may affect the results of the meta-analysis, we further analysed the judgement of the cut-off value, the source of the antibody and the technical method used in the eight studies (Supplementary Table 1). The study by Shi et al. (27) was excluded due to differences in the definition of the cut-off value. The study by Zhou et al. (31) was excluded because it did not clarify the specific cut-off value. The study by Xu et al. (30) was excluded as the cut-off value was defined as H-score ≥ 160, which was significantly higher than in other studies. The study by Wang et al. (28) was also excluded because c-MET expression was detected using the FISH method. We then performed a new meta-analysis after excluding these four studies, and the results suggested that patients with ESCC with high c-MET expression had a worse prognosis (HR = 1.54, 95% CI: 1.17–2.01; *p* = 0.002; Figure 3C).

### 3.4 Association between c-MET expression and clinicopathological parameters

**Figure 4:**
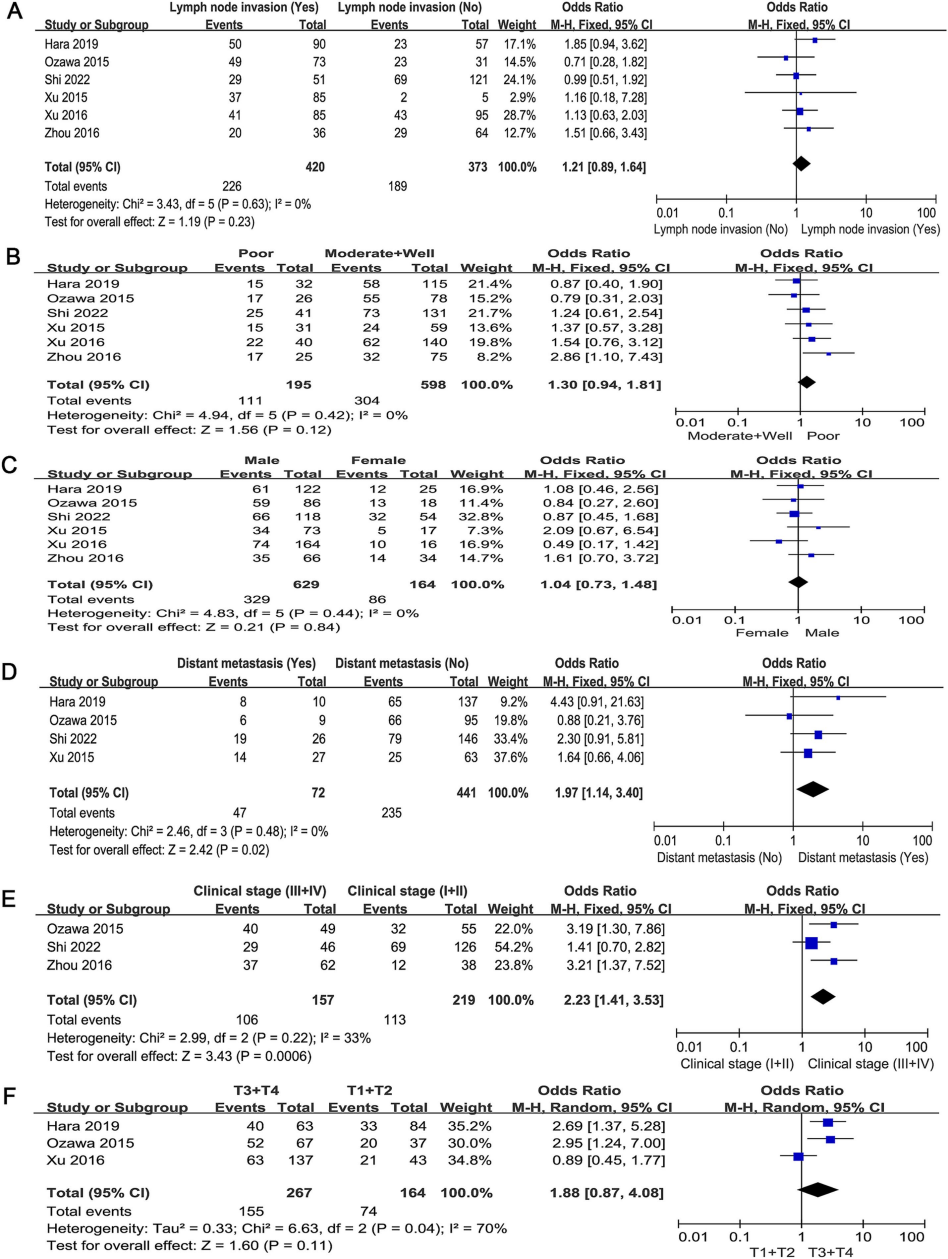
Forest plots of ORs for the association between c-MET expression and clinicopathological parameters in patients with ESCC. Forest plots for all data sets analysis based on **(A)** lymph node metastasis, **(B)** tumor differentiation, **(C)** gender, **(D)** distant metastasis, **(E)** clinical stage, and **(F)** T classification. ORs, odds ratios; c-MET, cellular-mesenchymal epithelial transition factor; ESCC, esophageal squamous cell carcinoma; CI, confidence interval.

Pooled ORs showed that c-MET expression was associated with distant metastasis (odds ratio [OR] = 1.97, 95% CI: 1.14–3.40; *p* = 0.02; Figure 4) and clinical stage (OR = 2.23, 95% CI: 1.41–3.53; *p* = 0.0006; Figure 4), although some missing information resulted in the inclusion of few studies. However, c-MET expression was not associated with lymph node metastasis (OR = 1.21, 95% CI: 0.89–1.64; *p* = 0.23; Figure 4), tumour differentiation (OR = 1.30, 95% CI: 0.94–1.81; *p* = 0.12; Figure 4), sex (OR = 1.04, 95% CI: 0.73–1.48; *p* = 0.21; Figure 4) or T classification (OR = 1.88, 95% CI: 0.87–4.08; *p* = 0.11; Figure 4). These results indicate that patients with ESCC with high c-MET expression are more likely to develop distant metastasis and experience accelerated tumour progression.

### 3.5 Heterogeneity analysis and publication bias

**Figure 5:**
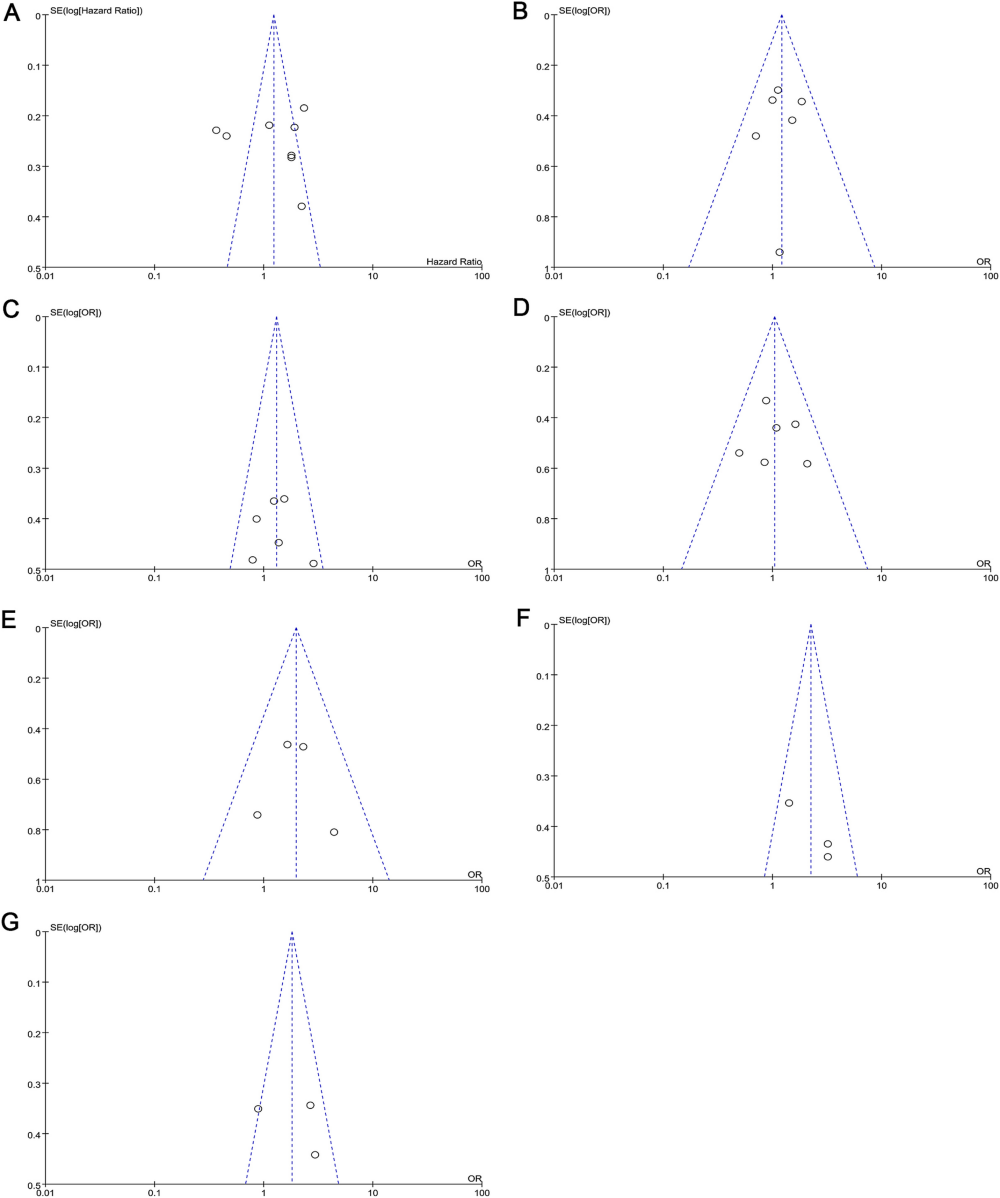
Funnel plots for the assessment of publication bias. Funnel plots for publication bias based on **(A)** OS, **(B)** lymph node metastasis, **(C)** tumor differentiation, **(D)** gender, **(E)** distant metastasis, **(F)** clinical stage, and **(G)** T classification. OS, overall survival.

To verify the robustness of the analytical results, we used the Q test and I^2^ statistic based on chi-squared statistics to assess heterogeneity across studies. Significant heterogeneity was detected when investigating the association between c-MET expression and tumour T stage, so we chose a random-effects model for analysis (*p* < 0.05; *I*^2^ = 70%). We also constructed Begg’s funnel plots to assess possible publication bias. Based on the shape of the funnel plot, we did not find any indication of publication bias in the meta-analysis (Figure 5). The results of Egger’s test were consistent with the observations from the funnel plots (Table 2).

**Table 2:** Results of Egger’s test.

Comparison	*t*	*p*-value	95%CI
OS	0.14	0.895	−14.713–16.4586
Lymph node metastasis	−0.30	0.777	−3.975–3.194
Tumor differentiation	0.30	0.778	−9.148–1.381
Sex	0.14	0.898	−5.839–6.441
Distant metastasis	0.12	0.914	−14.713–16.458
Clinical stage	4.74	0.132	−14.560–31.914
T classification	0.49	0.711	−150.95–163.00

OS, overall survival; CI, confidence interval.

## 4 Discussion

The c-MET protein, encoded by the MET proto-oncogene, possesses tyrosine kinase activity and functions as a receptor for HGF (15). In cellular function, c-MET plays a key role in regulating cell signalling and cytoskeletal reorganisation, processes essential for cell proliferation, differentiation and motility (32). Recently, with the development of targeted therapeutic strategies against c-MET, it has gained renewed attention due to its central role in tumour development. It has been reported that abnormal activation of c-MET can prompt normal cells to transform into tumour cells and further enhance the invasiveness, metastatic ability and spread of cancer cells (33, 34).

Given the important role of c-MET in tumour development, it has been extensively investigated as a prognostic indicator in a variety of cancers. Previous meta-analyses have shown that c-MET is a poor prognostic marker in head and neck squamous cell carcinoma (35, 36) and non-small cell lung cancer (37, 38). However, for ESCA, especially ESCC, there are few relevant studies, leaving a significant gap in understanding the prognostic value of c-MET in this type of cancer. Analysis of online data showed no significant association between c-MET mRNA levels in ESCA and overall prognosis, but there was an association with lymph node metastasis, tumour grade and stage. Considering that ESCC is the predominant subtype of oesophageal cancer in China (accounting for approximately 90%), we performed a meta-analysis to assess the prognostic and clinicopathological significance of c-MET protein expression in ESCC. Our findings showed that high c-MET expression was associated with a worse prognosis, an increased risk of distant metastasis and an advanced clinical stage in patients with ESCC. In this study, there was no statistically significant difference in OS between patients with high and low c-MET expression. We speculate that this may be due to the small sample size included in the database, which highlights the need for a larger sample size and more prognostic information for analysis.

Our study’s findings are consistent with existing evidence regarding c-MET overexpression in other cancer types. Specifically, c-MET overexpression is known to be prevalent in solid tumours, even when MET gene mutations or amplifications are rare (18, 28). For example, Liberati et al. (18) found that although MET mutations and amplifications are uncommon in ESCC (affecting only 5–6% of cases), 84% of cases exhibit at least a twofold increase in c-MET protein expression. Similarly, Wang et al. (28) reported that true MET amplification was present in only 1% of ESCC cases, whereas Hu et al. demonstrated frequent c-MET overexpression, particularly in well-to-moderately differentiated ESCC tumours. These findings align with our results and indicate that MET gene amplification is not the primary driver of c-MET overexpression in ESCC. Instead, c-MET protein upregulation may occur due to transcriptional mechanisms or post-transcriptional modifications, often following the activation of other driver genes that contribute to tumour progression. Although this study does not fully reveal the specific mechanisms underlying c-MET’s role in ESCC, it highlights c-MET’s significance in disease prognosis and the need for further research on its molecular pathways. Xu YP et al. (30) showed that OS was significantly different between patients with high MET expression and those with low or negative MET expression, and high MET expression was the only prognostic factor for OS. Shi Y et al. (27) reported that MACC1 may affect the prognosis of ESCC by regulating the expression of the MET/cyclin D1 axis. Yuan H et al. (39) reported that ISG15 promotes ESCC tumourigenesis via the MET/Fyn/*β*-catenin signalling pathway. However, the molecular mechanism of high MET expression in ESCC remains to be further investigated.

In the context of clinical applications, the findings reinforce c-MET’s potential as a valuable prognostic biomarker for ESCC. However, implementing c-MET as a routine prognostic marker or therapeutic target faces several challenges, including technical feasibility and cost-effectiveness, as c-MET testing often requires advanced, costly equipment and expertise. Furthermore, although c-MET-targeted therapies have shown promising results in other cancers, particularly non-small cell lung cancer (40), there remains a paucity of clinical trials and data on c-MET inhibitors in ESCC. Kashyap et al. (41) noted the absence of published clinical data on c-MET tyrosine kinase inhibitors for ESCC, emphasising a significant gap in translational research for this cancer subtype (41). Our study may provide foundational data to guide future clinical trials, potentially paving the way for ESCC-specific therapeutic strategies targeting c-MET.

It is important to acknowledge some limitations in our meta-analysis. This study included only four studies in the final analysis due to heterogeneity in cut-off values and detection methods, which may impact the robustness of our conclusions. Furthermore, some included studies had small sample sizes, which can influence the reliability of the results due to reduced statistical power. Larger, multicentre studies are needed to validate our findings and establish c-MET’s role more definitively in the prognosis of ESCC.

## 5 Conclusion

In conclusion, this study suggests that c-MET expression is a significant risk factor in ESCC; elevated c-MET levels are associated with poor survival outcomes, later clinical stages and increased distant metastasis. This knowledge may aid in identifying high-risk patients through c-MET expression assessment, thereby contributing to risk stratification efforts in ESCC. Based on these findings, c-MET expression holds promise as a prognostic marker in clinical practise, but further studies on c-MET in ESCC are warranted to solidify its role and advance therapeutic development. Future research should focus on larger sample sizes, exploration of molecular mechanisms and the assessment of clinical feasibility, including cost and accessibility, to ensure the efficient integration of c-MET as a prognostic tool in ESCC management.

## Data Availability

The original contributions presented in the study are included in the article/Supplementary material, further inquiries can be directed to the corresponding author.

## Footnotes

^1^ http://ualcan.path.uab.edu/

^2^ http://GEPIA.cancer-pku.cn/index.html
