# Supplementary material for: Prognostic value of c-MET in oesophageal squamous cell carcinoma: a study based on the mRNA expression in TCGA database and a meta-analysis
Source: Front Med (Lausanne). 2025 Feb 26;12:1548160. doi: 10.3389/fmed.2025.1548160 (PMC11897030; doi:10.3389/fmed.2025.1548160)
Supplement: Supplementary file 1 [file Table_1.doc]

**Supplementary Table 1. c-MET expression analysis from the 8 studies**

| **Studies** | **Antibody** | **Technology/H-score** | **Scoring system** |
| --- | --- | --- | --- |
| Hara 2019 | SP44 (1:400) | IHC/H-score ≥ 90 | Staining intensities were evaluated according to the following classification: 0, no staining; 1+ , weaker than the basal layer; 2+, the same intensity as the basal layer; 3+ , stronger than the basal layer. Staining proportions were determined as the percentage of positive cells with each intensity. Finally, we calculated an H-score by summing the values obtained when multiplying the intensities and proportions at each intensity. The cases scoring higher than the cut-off value, according to the median of each H-score, were c-MET positive, and the others were c-MET negative. |
| Kim 2016 | SP44 | IHC/H-score ≥ 50 | The c-MET staining intensity was scored as 0 (none or staining in less than 10% tumor cells), 1 (weak), 2 (moderate), or 3 (strong) based on membranous and/or cytoplasmic staining as previously reported, and each score multiplied by the percentage of cells (0%-100%). Therefore, H-score was ranged from 0 to 300. The median value 50 of c-MET H-score among samples with positive c-MET IHC staining was arbitrarily defined as the cut-off value for c-MET positive. |
| Ozawa 2015 | IBL, Japan (1:50) | IHC/H-score ≥ 40 | Immunohistochemistry was evaluated and scored according to the following criteria: 0, completely negative; 1+, weakly positive; 2+, moderately positive, and 3+, markedly positive. The H-score was then calculated by multiplying the percentage of positive cells to the score (H-score ranging from 0 to 300). |
| Shi 2022 | Bioss, China, (1:300) | IHC/IHC Score > 4 | Staining intensities: “0” for no color, “1” for light yellow, “2” for yellow, and “3” for brown. The percentage of positive cells was calculated under the view, and scoring was performed according to the following standards: ≤5%, a score of “0”; 6–25%, a score of “1”; 26– 50%, a score of “2”; and 51–100%, a score of “3.” The final score was obtained by multiplying the average staining intensity of each slice by the average percentage of positive cells, with 0–4 score for negative (−) and 5–9 for positive (+). |
| Wang 2019 | NA | FISH | An average MET gene copy number ≥ 5 and a MET/CEP7 ratio ≥ 2 (true MET amplification) were regarded as MET FISH positive. |
| Xu 2015 | SP44 | IHC/H-score ≥ 20 | Intensity was scored according to a four-tier systems: 0, no staining; 1+, weak; 2+, moderate; and 3+, strong. H-score assessment was based on staining intensity (0–3) and the percentage of positive cells (0-100%). Each individual intensity level was multiplied by the percentage of cells and all values were added to obtain. The final IHC score, ranging from 0 to 300. The final score was calculated from the scores of assessment at membranous and cytoplasmic expression. |
| Xu 2016 | EP1454Y | IHC/H-score ≥ 160 | H-score assessment was based on a combination of the percentage and the intensity of the stained tumor cells. Each individual intensity level (0–3) was multiplied by the percentage of cells(0%-100%), and all values were added to generate the final IHC score, with an H-score range of 0 to 300. |
| Zhou 2016 | CST | IHC/NA | H-score assessment was based on a combination of the percentage and the intensity of the stained tumor cells. |

Abbreviations: IHC, immunohistochemistry; NA, not availiable; FISH, Fluorescence in situ hybridization.
